# Construction of a readmission prediction model and development of a decision support system for chronic heart failure patients based on patient-reported outcome measures

**DOI:** 10.3389/fmed.2026.1748709

**Published:** 2026-09-23

**Authors:** Chen Yu, Miaomiao Lou, Yang Sun, Zhe Yang, Liang Zhu

**Affiliations:** 1 Health Service Management and Medical Education, School of Preventive Medicine, The Fourth Military Medical University, Xi'an, Shaanxi, China; 2 The Ministry of Education Key Lab of Hazard Assessment and Control in Special Operational Environment, Xi'an, China; 3 The Shaanxi Provincial Key Laboratory of Environmental Health Hazard Assessment and Protection, Xi'an, China; 4 Information Department, Xijing Hospital, Fourth Military Medical University, Xi'an, Shaanxi, China; 5 Department of Geriatrics, Xijing Hospital, Fourth Military Medical University, Xi'an, Shaanxi, China; 6 Department of Health Statistics, School of Preventive Medicine, The Fourth Military Medical University, Xi'an, Shaanxi, China

**Keywords:** automated machine learning, biopsychosocial model, decision support system, heart failure readmission, patient-reported outcome measures, SHAP explainability analysis

## Abstract

**Objective:**

To construct a readmission risk prediction model for heart failure based on an Automated Machine Learning (AutoML) framework and develop a Clinical Decision Support System (CDSS) integrating Patient-Reported Outcome Measures (PROM), aiming to address the limitations of traditional models in processing multidimensional physiological-psychological-social data and their low clinical translation efficiency.

**Methods:**

A retrospective cohort study design was employed, enrolling 552 chronic heart failure patients (training-to-testing set ratio of 8:2). Multidimensional features were collected via electronic medical records and the Chinese version of the Heart Failure-Specific PROM Scale (CHF-PROM). Missing data were handled using Multivariate Imputation by Chained Equations (MICE). An AutoML framework driven by an Improved Pied Kingfisher Optimizer (IPKO) algorithm, integrating algorithms including Logistic Regression, Support vector machine (SVM), XGBoost, and LightGBM, was developed. Model performance and interpretability were evaluated using the Area Under the Receiver Operating Characteristic Curve (AUC), Decision Curve Analysis (DCA), and SHapley Additive exPlanations (SHAP). A visualized decision-making tool was implemented on the MATLAB platform.

**Results:**

Testing set showed the AutoML model achieved significantly enhanced predictive efficacy [AUC = 0.8909, 95% Confidence Interval (CI): 0.8392 to 0.9426]. SHAP analysis revealed key multidimensional factors influencing prognosis, among which milder depressive symptoms, higher levels of social support, and favorable appetite-sleep status were important protective factors reducing readmission risk, while female sex and advanced age were identified as risk predictors. Decision curve analysis further confirmed that applying this model yielded positive net clinical benefit across a wide risk threshold range of 1.2% to 78.6%.

**Conclusion:**

This study achieved efficient translation of PROM data into clinical decisions and innovatively demonstrated the independent predictive value of psychosocial factors for readmission. The developed CDSS system provides a feasible pathway for the proactive prevention and management of heart failure.

## 1 Introduction

Chronic heart failure (CHF), as the end-stage manifestation of cardiovascular disease characterized by high morbidity and disability rates, has become a significant global public health burden. According to statistics from the World Health Organization, the number of CHF patients worldwide has exceeded 26 million and continues to rise steadily due to the aging population (1). More critically, readmission rates among CHF patients remain persistently high. Data from developed countries indicate that approximately 25% of patients are readmitted within 30 days of discharge due to disease exacerbation, while the 90-day readmission rate soars to 30%−50% (2, 3). Readmission events not only serve as warning signs of disease progression but also contribute to a vicious cycle involving increased all-cause mortality, significantly worsened quality of life, and soaring healthcare expenditures (4, 5). This stark reality underscores the deficiencies in existing clinical management strategies regarding dynamic risk identification and early intervention mechanisms.

Traditional readmission risk prediction models primarily depend on clinical indicators (such as left ventricular ejection fraction, BNP levels, renal function parameters) and demographic characteristics recorded at discharge (6, 7). The limitations of these models are increasingly apparent: firstly, the static recording of biomedical indicators fails to capture the dynamic physiological changes patients experience post-discharge. Secondly, they neglect the independent impact of patient subjective health experiences and functional status on prognosis. Thirdly, traditional statistical methods like linear regression struggle to handle complex nonlinear interactions among variables (8, 9). Notably, recent research evidence demonstrates that health-related quality of life (HRQOL), a core measure of patients' multidimensional health perception, holds significant predictive value for cardiovascular events. A scientific statement from the American Heart Association (AHA) explicitly states: “Patient-reported problems in quality-of-life domains, such as physical functioning limitations, emotional distress, and social role limitations, predict heart failure readmission risk independently of traditional risk factors. For every 5-point decrease in HRQOL, the rehospitalization risk increases by 12%” (10–12).

Against this backdrop, Patient-Reported Outcome Measures (PROM) provide a novel perspective to overcome the limitations of traditional prediction models, serving as the standard tool for quantifying HRQOL (13, 14). PROM systematically evaluates patients' self-perceived status across dimensions such as physical function, symptom burden, emotional wellbeing, and social participation through structured scales. Its unique value manifests in three aspects: (1) Individualized Dynamic Capture: Compared to single clinical measurements, PROM can continuously track the evolution of functional status post-discharge, revealing compensatory disability processes not reflected by clinical indicators; (2) Complement to Clinical Decision-Making: Patient-reported psychosocial stressors (e.g., depression and anxiety) have been shown to accelerate heart failure progression through neuroendocrine activation, yet are often underestimated in physician-patient communication; (3) Predictive Performance Optimization: Prospective cohort studies incorporating PROM instruments like the Kansas City Cardiomyopathy Questionnaire (KCCQ) have confirmed that PROM scores can achieve an area under the receiver operating characteristic curve (AUC) of 0.74 for predicting readmission within 3 months, significantly higher than models solely based on biomarkers. However, the translation of PROM into clinical practice faces two major challenges: On one hand, the heterogeneous data generated by multidimensional items encompass substantial nonlinear relationships and interaction effects, which traditional logistic regression struggles to fully exploit for their informational potential. On the other hand, there is a lack of integrated solutions for transforming PROM-based predictions into visualized clinical decision support tools.

In recent years, the application of Machine Learning (ML) technology in healthcare prediction has offered a new paradigm for processing high-dimensional, heterogeneous data. Ensemble tree models can effectively identify latent interaction patterns among PROM items through multi-level variable splitting strategies; neural networks can model highly nonlinear feature responses (15, 16). However, conventional ML workflows are highly dependent on the parameter tuning and feature engineering expertise of professionals, creating high barriers to clinical application. The advent of Automated Machine Learning (AutoML) frameworks provides a breakthrough path to this challenge, systematically addressing the complexities of feature selection, algorithm selection, and parameter tuning through meta-learning, Bayesian optimization, and automated feature engineering (17).

Based on the aforementioned background, this study proposes an innovative research framework that integrates the value propositions of PROM with the advantages of artificial intelligence technology. The objectives are to: (1) construct a readmission prediction model for heart failure based on patients' subjective health perceptions, and (2) develop an interactive Clinical Decision Support System (CDSS). Through visualized risk stratification and personalized management modules, the framework aims to achieve a closed-loop transformation from prediction algorithms to bedside intervention. The overarching goal is to advance the practical application of AI-assisted decision-making in chronic disease management and explore the feasibility of leveraging digital health tools to optimize the distribution of medical resources.

## 2 Methods

### 2.1 Study design and participants

This retrospective cohort study systematically enrolled CHF patients admitted to Xijing Hospital, The Fourth Military Medical University, between May 2021 and October 2023. A total of 552 participants were included based on predefined eligibility criteria. As a retrospective study requiring no informed consent was waived by the Ethics Committee of Xijing Hospital (Approval No.: KY20262037-F-2), the research was conducted in accordance with the ethical standards outlined in the World Medical Association Declaration of Helsinki.

Inclusion criteria: Age ≥18 years; Meeting diagnostic criteria for New York Heart Association (NYHA) functional class II-IV; Receiving standardized heart failure (HF) pharmacotherapy during hospitalization.

Exclusion criteria: History of acute cardiovascular events (including myocardial infarction, stroke, or unstable angina) within 2 months preceding the final follow-up; Presence of non-HF-related high-risk factors for readmission such as malignancy or end-stage renal disease.

From May 2021 to October 2023, a total of 823 hospitalized patients with chronic heart failure were consecutively screened. After excluding 271 patients who did not meet the inclusion criteria, including those with acute cardiovascular events (within 2 months), end-stage renal disease, malignancy, and other conditions, 641 patients were enrolled in the prospective follow-up cohort. During the 12-month follow-up period, 89 patients with NYHA class IV (accounting for 23.7% of that class) failed to complete all PROM assessments and endpoint determination due to severe physical or cognitive impairment caused by disease progression (47 cases), refusal to continue participation (23 cases), loss to follow-up due to change of contact information (12 cases), or death (7 cases), and were therefore excluded. The final analysis cohort comprised 552 patients who completed all follow-ups and had complete readmission outcome information, with an all-cause readmission incidence rate of 33.33% (184/552). The specific flow and reasons at each stage are detailed in Figure 1.

**Figure 1:**
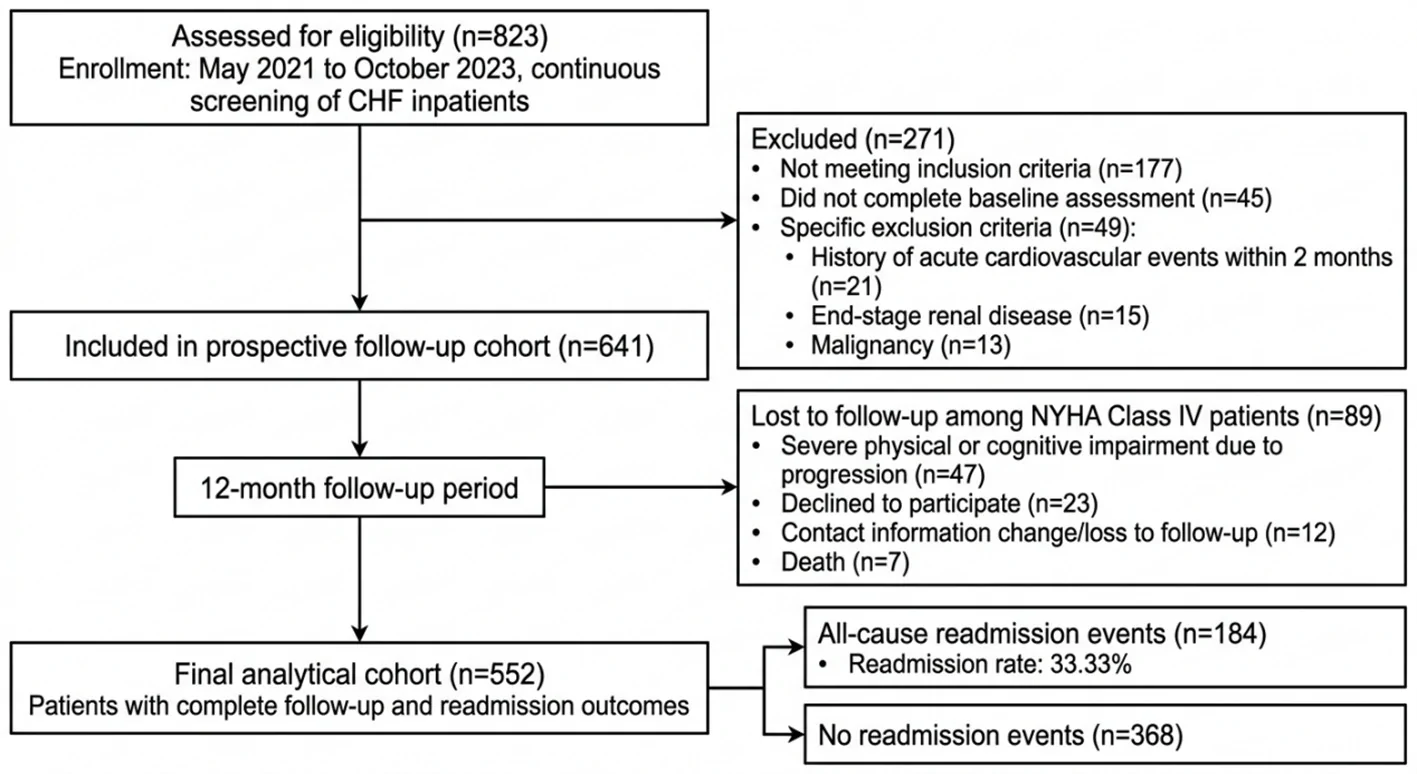
Patient enrollment and follow-up flowchart.

Definition of all-cause readmission: any rehospitalization event within 12 months after discharge for any reason (cardiac or non-cardiac), excluding cases involving only emergency department visits without formal admission procedures.

### 2.2 Data collection and processing

Research data were collected through electronic medical record (EMR) systems and structured questionnaires, with verification performed by two certified researchers to ensure accuracy. Baseline data encompassed: Clinical characteristics: Age, sex, smoking history, alcohol consumption, body mass index (BMI), and NYHA classification; CHF-specific Patient-Reported Outcome Measures (CHF-PROM): Health-related quality of life (HRQOL) was assessed using the validated Chinese version of the Chronic Heart Failure Patient-Reported Outcome Scale (14). Developed based on the biopsychosocial medical model, this scale encompasses four core domains (physical, psychological, social, and therapeutic) comprising 12 subdimensions and 57 items (Supplementary Table 1). Items were scored on a 5-point Likert scale, with raw scores ranging from 0 (“Never”) to 4 (“Always”). To mitigate response bias from reverse-worded items, standardized directional adjustment was applied: positively worded items retained raw scores, while negatively worded items underwent linear transformation (adjusted score = 4–raw score). This procedure ensured uniform scaling (0–4) across all 57 valid items, establishing a positive correlation between total score and health status (higher total scores indicating improved HRQOL); Clinical Outcome: The primary endpoint was all-cause readmission (yes/no). All patients underwent routine 12-month post-discharge follow-up. Readmission events were verified through outpatient reviews, telephone follow-ups, and EMR audits.

To reduce social desirability response bias, all PROM assessments were completed under the guidance of trained research nurses in an independent, quiet room, using standardized instructions that emphasized there were no right or wrong answers, complete confidentiality, and that the results would be used solely for scientific research. In addition, we performed directional adjustment on all items (negatively worded items were reverse-scored into positive scores) and examined the internal consistency of each case, with no obvious extreme response patterns identified.

The missing proportion for each variable in the dataset was below 5%, with missing data concentrated primarily in PROM dimensions involving socially sensitive health self-assessments such as anxiety, depression, and sleep. Little's MCAR test and visual pattern exploration confirmed that the missing mechanism conformed to the missing-at-random assumption. To prevent the testing set information from interfering with the imputation process, the following strategy was adopted in this study: first, the training set and testing set were divided using the outcome variable as the stratification factor; then, a multivariate imputation by chained equations model was constructed on the training set, incorporating all PROM items, the all-cause readmission outcome, and key covariates including age, sex, NYHA classification, and number of comorbidities, with 50 iterations performed to generate 50 complete training set datasets. The imputation parameters fitted on the training set were used to perform unidirectional multiple imputation of missing values for the testing set, producing 50 complete testing set datasets. The final model evaluation used the first complete dataset from the 50 imputations for each testing set sample, ensuring that the testing set imputed values were completely independent of the testing set's own outcome information. Imputation stability was comprehensively evaluated within the training set: the standardized root mean square error across all variables was 0.062, and all variance inflation factors were below 5. Table 1 reports the fraction of missing information for the main variables in the training set, where the lowest FMI was 0.07 (suspiciousness dimension) and the highest was 0.18 (appetite-sleep dimension); within this range, the actual relative efficiency of 50 imputations exceeded 99.6% for all variables.

**Table 1:** Fraction of missing information (FMI) for main predictor variables.

Variable	Missing proportion	FMI
Depression dimension	3.2%	0.12
Anxiety dimension	2.8%	0.10
Social support dimension	4.1%	0.15
Suspiciousness dimension	1.9%	0.07
Appetite-sleep dimension	4.6%	0.18
NYHA classification	0%	—

### 2.3 Adaptive machine learning model construction

This study constructed an automated machine learning framework based on a hybrid metaheuristic-driven parameter optimization engine to systematically handle base learner selection, feature screening, and hyperparameter tuning (18). All modeling work was carried out in the MATLAB R2024a environment. To ensure full reproducibility of the entire process, fixed random number generator seeds were set for all procedures involving randomness: the dataset was split via stratified random sampling using the primary outcome variable (whether all-cause readmission occurred) as the stratification factor, generating a training set (*n* = 423) and an internal testing set (*n* = 129) at an 8:2 ratio, with a fixed random seed of 42; the readmission positive rate was 33.33% in both the training and validation sets (141 cases in the training set, 43 cases in the validation set); the population initialization process in the IPKO algorithm used a seed value of 123. The core components of the framework included: first, an Improved Pied Kingfisher Optimizer (IPKO), which builds upon the Pied Kingfisher Optimizer by reconstructing the initial population distribution through sinusoidal chaotic mapping to prevent premature convergence (19), and embedding a dynamic Gaussian walk strategy to adaptively balance global exploration and local exploitation capabilities, with its complete pseudocode provided in Supplementary Material 1; second, an integrated predictive architecture, where the cohort was randomly partitioned into a training set and an internal testing set, and the baseline machine learning models included logistic regression, support vector machine, and the gradient boosting frameworks XGBoost and LightGBM, which were dynamically integrated by the AutoML framework using IPKO.

To ensure complete transparency of the model construction process, the hyperparameter space searched by the IPKO algorithm for each base learner and the final selected optimal configurations are detailed in Table 2. All continuous predictor variables were subjected to Z-score normalization before being input into the models. For multi-category variables, NYHA functional classification was encoded using dummy variable coding, i.e., converted into k-1 binary variables entering the models, to eliminate baseline bias and ensure independent estimation of each category's effect.

**Table 2:** Hyperparameter search space and optimal parameter configuration in IPKO-AutoML.

Base learner	Hyperparameter	Search range	Step or value method	Final selected value
Logistic regression	Regularization strength C	0.01 to 100	logarithmic scale	1.23
SVM	Penalty coefficient C	0.1 to 100	logarithmic scale	10.5
	Kernel coefficient γ	0.001 to 10	logarithmic scale	0.15
XGBoost	Learning rate	0.01 to 0.3	interval 0.01	0.12
	Max tree depth	3 to 10	interval 1	6
	Subsample ratio	0.6 to 1.0	interval 0.1	0.8
LightGBM	Subsample ratio	0.01 to 0.3	interval 0.01	0.08
	Number of leaves	20 to 100	interval 5	55
	Feature sampling ratio	0.5 to 1.0	interval 0.1	0.9

In this study, the proportion of positive events was 33.33%, constituting a mild-to-moderate class imbalance. To make the model pay sufficient attention to the minority class during training, we enabled the cost-sensitive learning parameter scalefor XGBoost and LightGBM within the AutoML hyperparameter search space, with the search range set to [1, number of negative cases / number of positive cases in the training set]. Through automatic selection via Bayesian optimization, the optimal value of this parameter was determined to be 2.0, which is exactly consistent with the negative-to-positive ratio in the training set. In addition, model performance evaluation used the F1-score and area under the precision-recall curve as the core discriminative metrics; these two metrics are significantly more sensitive to class imbalance than overall accuracy, and can more truly reflect the model's ability to identify patients at high risk of readmission. This modeling did not employ resampling strategies such as oversampling or undersampling, to avoid the risk of overfitting or loss of effective information that may be caused by artificially synthesized samples.

### 2.4 Evaluation metrics

A multidimensional evaluation system was established: (1) Discriminatory Performance: Prognostic prediction models were assessed using six core metrics: Accuracy (ACC), Sensitivity (SEN), Specificity (SPE), F1-score (harmonic mean of precision and recall), Area Under the Receiver Operating Characteristic Curve (AUC), and Area Under the Precision-Recall Curve (AUPRC) to systematically evaluate model discrimination and robustness under class imbalance; (2) Calibration Performance: Calibration curves complemented by the Brier score (lower values indicate greater predictive accuracy) assessed probabilistic prediction calibration; (3) Clinical Utility: Decision Curve Analysis (DCA) quantified clinical net benefit (NB) across threshold probabilities, calculated as:


$$\begin{array}{l} NB=\frac{TP}{N}-\frac{FP}{N}\times \frac{P_{t}}{1-P_{t}} \end{array}$$

where *TP* is true positives, *FP* is false positives, *N* is total sample size, and pt is the risk threshold. By comparing *NB* with reference lines for traditional intervention strategies, the effective interval for model-assisted decision-making was validated.

To comprehensively evaluate the predictive accuracy of the model, in addition to discrimination metrics, this study employed calibration intercept and calibration slope to assess the systematic bias and degree of overfitting of the model's probability predictions, in accordance with the TRIPOD-AI reporting guidelines. The calibration intercept reflects the “calibration-in-the-large,” with an ideal value of 0; the calibration slope reflects the degree of shrinkage in the predictive effect, with an ideal value of 1. Simultaneously, the Brier score (range 0–1, with lower values indicating more accurate probability predictions) was reported to comprehensively measure both discrimination and calibration. To obtain a more realistic estimate of model performance approximating external generalizability and to quantify the optimism bias arising from the model's overadaptation to the training data, we performed internal testing and correction of the optimism estimate for the AUC of the final model on the training set using the 1,000-iteration Bootstrap resampling method. Specifically, for each bootstrap iteration, a new training set of the same sample size was generated by drawing with replacement from the original training set, and the AutoML model was refitted on this set; the AUC was then calculated separately on the training set of the current resample and on the out-of-bag samples not selected in that draw. The average of the difference between these two AUCs yielded the single-iteration optimism estimate, and the final corrected value was obtained by averaging over 1,000 iterations.

### 2.5 Explainability analysis

A dual-path validation framework integrating machine learning and classical statistics analyzed prediction mechanisms. Firstly, the Shapley Additive exPlanations (SHAP) method based on game theory quantified feature contributions. Global feature importance rankings revealed overall variable impact, while local contribution visualizations per sample enabled “macro-micro” cascade analysis of decision logic. Secondly, selected variables were incorporated into multivariate logistic regression for risk validation, with odds ratios (ORs) and 95% confidence intervals (CIs) computed to evaluate independent associations. The synergistic application leveraged SHAP's capacity for nonlinear interaction decomposition and the parametric explicability of traditional regression, ensuring bidirectional mapping between high-dimensional modeling outcomes and clinical pathological mechanisms. This approach maintained complex data adaptability while meeting evidence-based causal interpretation requirements.

### 2.6 Interactive tool development

An interactive clinical decision support system (CDSS) was developed using MATLAB 2024a App Designer. This software integrated the deployed machine learning model to assist clinicians in assessing CHF patient readmission risk through an intuitive graphical interface. The design encompassed three functional units: (1) Risk Stratification Module: visualized individual patient risk profiles with SHAP waterfall charts; (2) Personalized Intervention Recommender: Generated evidence-based management plans mapped to key contributing factors; (3) Population Health Dashboard: Aggregated cohort-level metrics to support resource allocation decisions.

### 2.7 Statistical analysis

Data were processed using SPSS 26.0. Normally distributed continuous variables were expressed as mean ± standard deviation (x ± s), non-normally distributed continuous variables as median [Q1, Q3], and categorical variables as frequencies and percentages [n (%)]. Intergroup comparisons for continuous variables employed the independent samples *t*-test for normally distributed data and the Mann-Whitney U test for non-normal distributions. Categorical variable comparisons used Pearson's chi-square test. All comparisons were two-sided with a significance threshold of α = 0.05, and results were presented in structured tables.

## 3 Results

### 3.1 Study cohort characteristics

Among the 552 patients, 184 (33.33%) experienced readmission. Clinical characteristics of the training set (*n* = 423) and testing set (*n* = 129) were comparable, with no statistically significant differences observed across all variables (*p* > 0.05, Table 3).

**Table 3:** Comparison of clinical characteristics between training set and testing set.

Variable	Training set (*n* = 423)	Testing set (*n* = 129)	Statistic	*P*-value
Age (years), mean ± SD	68.29 ± 11.43	67.55 ± 12.05	0.636	0.525
Sex, *n* (%)			0.649	0.420
Male	240 (56.74)	68 (52.71)		
Female	183 (43.26)	61 (47.29)		
BMI (kg/m^2^), mean ± SD	20.49 ± 3.46	21.18 ± 3.82	1.961	0.054
Smoking history, *n* (%)			0.018	0.893
Yes	122 (28.84)	38 (29.46)		
No	301 (71.16)	91 (70.54)		
Alcohol consumption, *n* (%)			0.108	0.743
Yes	128 (30.26)	41 (31.78)		
No	295 (69.74)	88 (68.22)		
NYHA classification, *n* (%)			0.690	0.708
ClassII	128 (30.26)	42 (32.56)		
ClassIII	168 (39.72)	46 (35.66)		
ClassIV	127 (30.02)	41 (31.78)		

### 3.2 Algorithm enhancement performance

To systematically evaluate the optimization capability of the Improved Pied Kingfisher Optimizer (IPKO), we conducted multidimensional comparative experiments against the original PKO and three novel intelligent algorithms [Ant Lion Optimizer (ALO), Harris Hawks Optimization (HHO), Whale Optimization Algorithm (WOA)]. Using 12 benchmark functions from the CEC2022 test suite under standard evaluation criteria (decision space dimension D = 10, population size *N* = 30, maximum iterations T= 500), each algorithm underwent 30 independent random-initialization runs. Quantitative results (Figure 2A) demonstrated IPKO outperformed all comparators in 91.67% (11/12) of test functions, exhibiting superior stability. Convergence curve analysis (Figure 2B) confirmed IPKO achieved faster convergence with minimal local optima entrapment risk, validating its enhanced global optimization efficiency.

**Figure 2:**
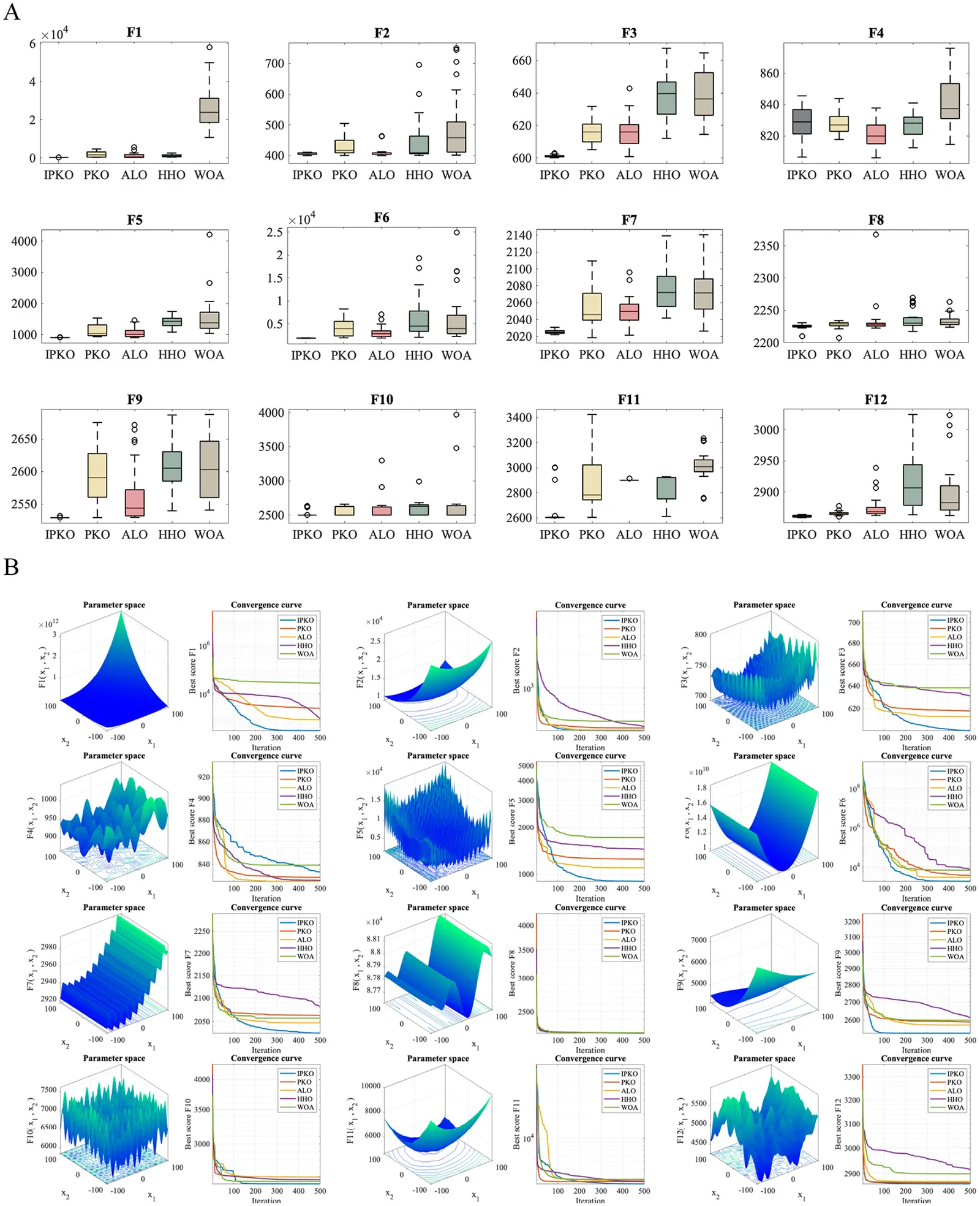
Comparative optimization performance of swarm intelligence algorithms. **(A)** Box plots display optimization results from 30 independent runs per algorithm on CEC2022 test functions, illustrating optimization stability and robustness; **(B)** Convergence curves reflect convergence speed and local optimum avoidance capability during optimization.

### 3.3 Model training outcomes

Feature selection via the IPKO-AutoML framework identified seven key predictors: sex, age, depression, anxiety, social support, suspiciousness, and appetite sleep. The framework autonomously selected LightGBM as the optimal base learner. Cross-validation demonstrated IPKO-AutoML's significant performance advantage over five traditional models, achieving an F1-score of 0.7794, ROC-AUC of 0.9392, and PR-AUC of 0.8573 (Table 4, Figure 3).

**Table 4:** Cross-validation performance in training set.

Models	PRE	SEN	SPE	ACC	F1	ROC-AUC (95% CI)	PR-AUC
LR	0.4670	0.6589	0.6701	0.6667	0.5466	0.7047 (0.6528–0.7566)	0.5252
SVM	0.5928	0.7674	0.7687	0.7683	0.6689	0.8116 (0.7691–0.8541)	0.6267
XGBoost	0.6024	0.7752	0.7755	0.7754	0.6780	0.8176 (0.7753–0.8599)	0.6902
LightGBM	0.6797	0.8062	0.8333	0.8251	0.7376	0.8244 (0.7825–0.8663)	0.6757
AutoML	0.7413	0.8217	0.8741	0.8582	0.7794	0.9392 (0.9148–0.9636)	0.8573

CI, confidence interval. The 95% confidence interval for ROC-AUC was calculated using the DeLong method.

**Figure 3:**
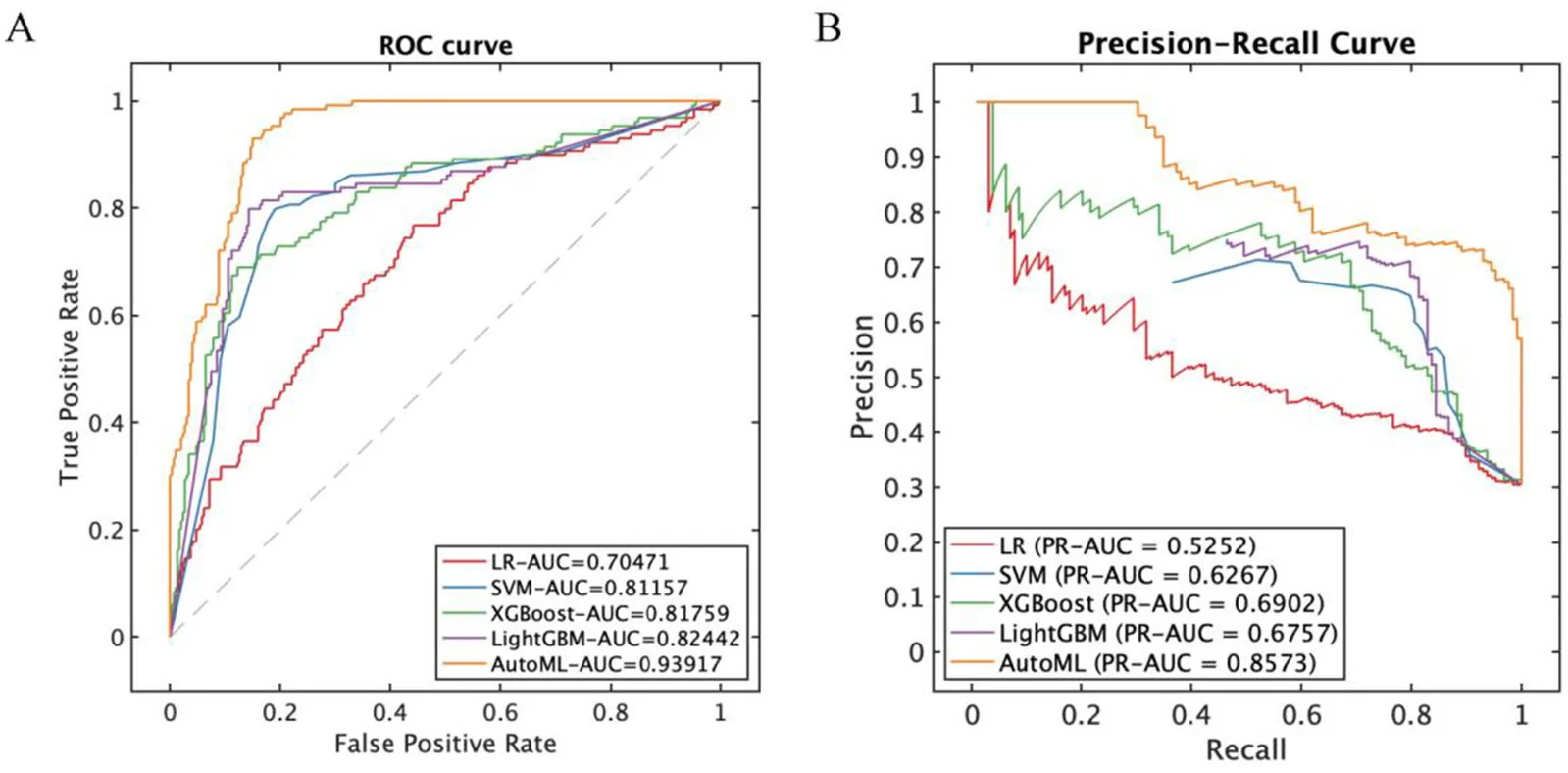
Cross-validation performance in training set. **(A)** ROC curve for training set; **(B)** PR curve for training set.

### 3.4 Predictive performance validation

Evaluation results on the testing set further validated the optimal predictive capability and robust generalization performance of the AutoML model, achieving an ROC-AUC of 0.8909 (95% CI: 0.8392–0.9426). The model's F1 score was 0.7429, and the area under the precision-recall curve (PR-AUC) was 0.8106, demonstrating outstanding precision and specificity while maintaining high sensitivity, thereby reflecting good comprehensive diagnostic value (Table 5, Figure 4).

**Table 5:** Performance metrics in testing set.

Models	PRE	SEN	SPE	ACC	F1	ROC-AUC (95% CI)	PR-AUC
LR	0.3846	0.5556	0.5429	0.5472	0.4545	0.6064 (0.5050–0.7078)	0.4167
SVM	0.5000	0.5556	0.7143	0.6604	0.5263	0.6357 (0.5364–0.7350)	0.4756
XGBoost	0.6098	0.6944	0.7714	0.7453	0.6494	0.7353 (0.6447–0.8259)	0.5257
LightGBM	0.6585	0.7500	0.8000	0.7830	0.7013	0.7829 (0.6975–0.8683)	0.6144
AutoML	0.7647	0.7222	0.8857	0.8302	0.7429	0.8909 (0.8392–0.9426)	0.8106

CI, confidence interval. The 95% confidence interval for ROC-AUC was calculated using the DeLong method.

**Figure 4:**
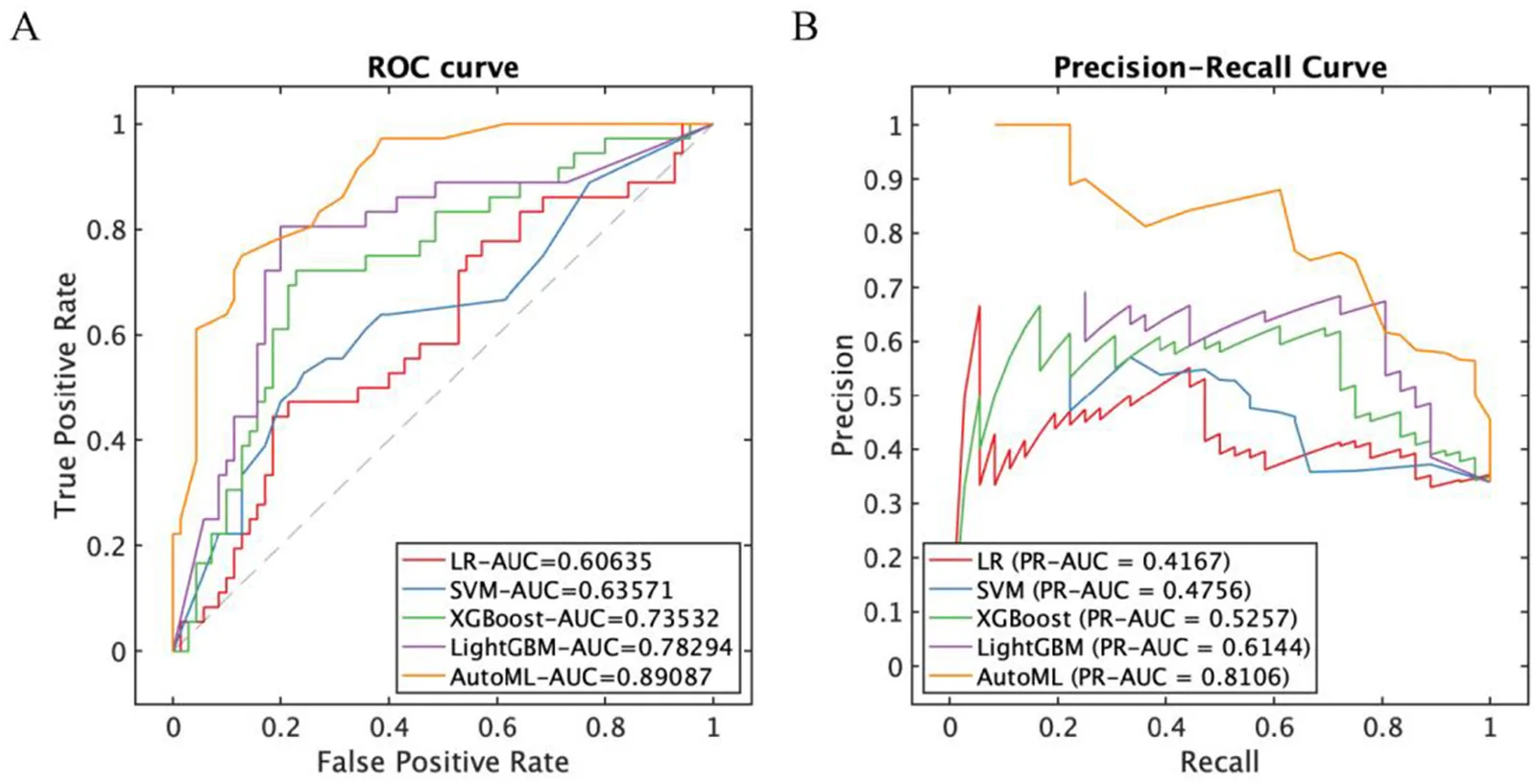
Performance metrics in testing set. **(A)** ROC curve for testing set; **(B)** PR curve for testing set.

On the independent testing set, the probability predictions of the AutoML model exhibited satisfactory calibration. The model's Brier score was 0.142, indicating a high degree of consistency between predicted risk and the actual incidence of readmission. The calibration intercept was −0.035 (95% CI: −0.291 to 0.221; *P* = 0.786), which was not statistically significant, suggesting no substantial mean bias in the model's predicted probabilities; the calibration slope was 0.951 (95% CI: 0.868–1.034), very close to the ideal value of 1, indicating minimal shrinkage in the predictive effect and no overt overfitting. To address the potential optimism bias arising during the training process, the corrected AUC of the model was adjusted from the original 0.9392 to 0.928 after calibration using 1,000 Bootstrap resamples. Although this corrected value slightly declined compared to the original AUC on the training set, it remained at an excellent level, providing a more conservative reflection of the model's generalizability potential in similar populations.

### 3.5 Clinical utility assessment

Decision curve analysis demonstrated that applying the intervention strategy based on the AutoML model yielded a positive net benefit across the entire clinically relevant high-risk threshold interval (1.2%−78.6%), with its curve lying consistently above the two extreme strategy lines of “treat all” and “treat none”. This finding suggests that using this model to assist clinical decision-making has the potential to bring benefits to patients over a broader risk range compared with conventional approaches; however, the specific magnitude of attributable benefit still requires confirmation in future multicenter prospective validations (Figure 5).

**Figure 5:**
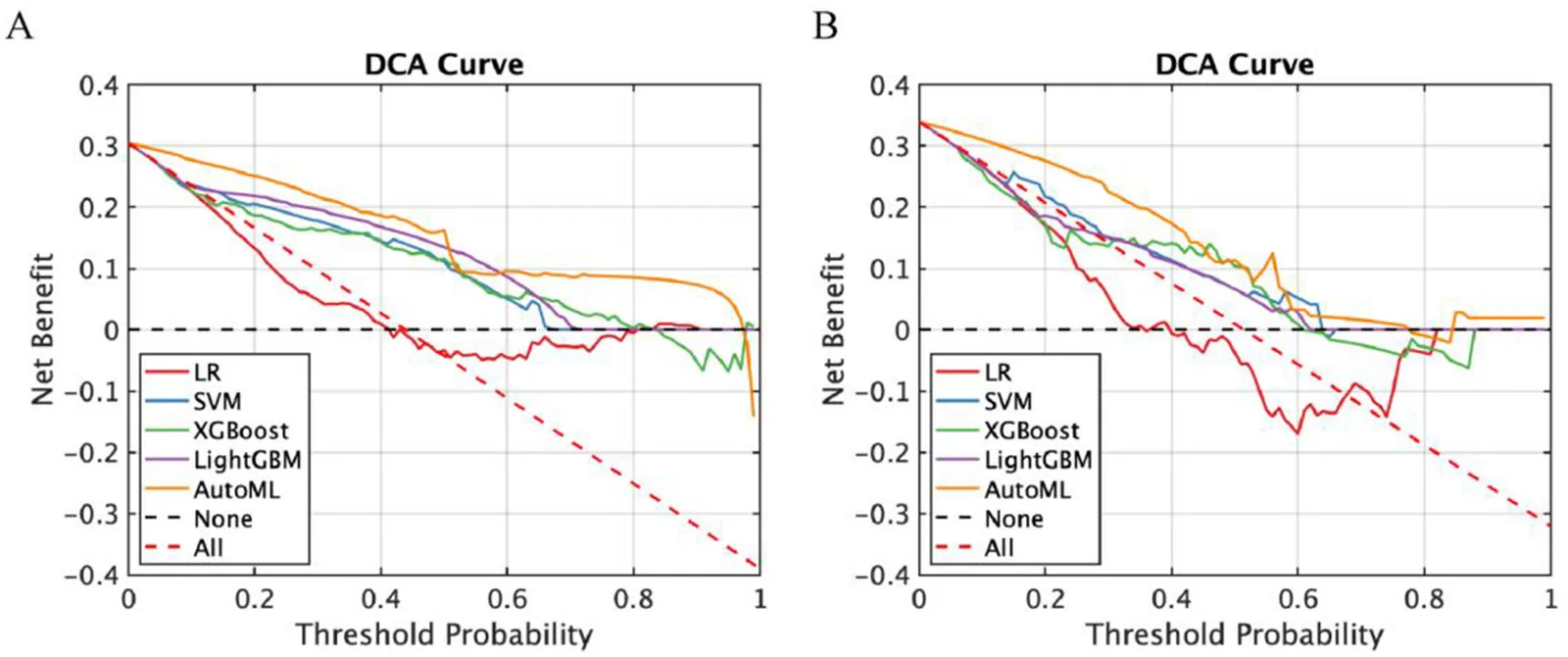
Decision curve analysis of prediction models. **(A)** Training set; **(B)** Testing set.

### 3.6 Interpretability analysis

SHAP analysis identified feature importance ranking: sex > age > depression > anxiety > social support > suspiciousness > appetite sleep (Figure 6). SHAP interaction plots displayed (Figure 7): (A) Dual-high risk: Patients ≥70 years with depression scores < 12 (red zone) showed elevated readmission risk; (B) Anxiety moderation: High social support amplified anxiety-reduction benefits (blue diamonds); (C) Age-specific protection: Social support demonstrated enhanced protective effects in elderly patients (red diamonds); (D) Triple protection: High appetite sleep combined with high depression scores (blue diamonds) indicated synergistic physiological-psychological protection.

**Figure 6:**
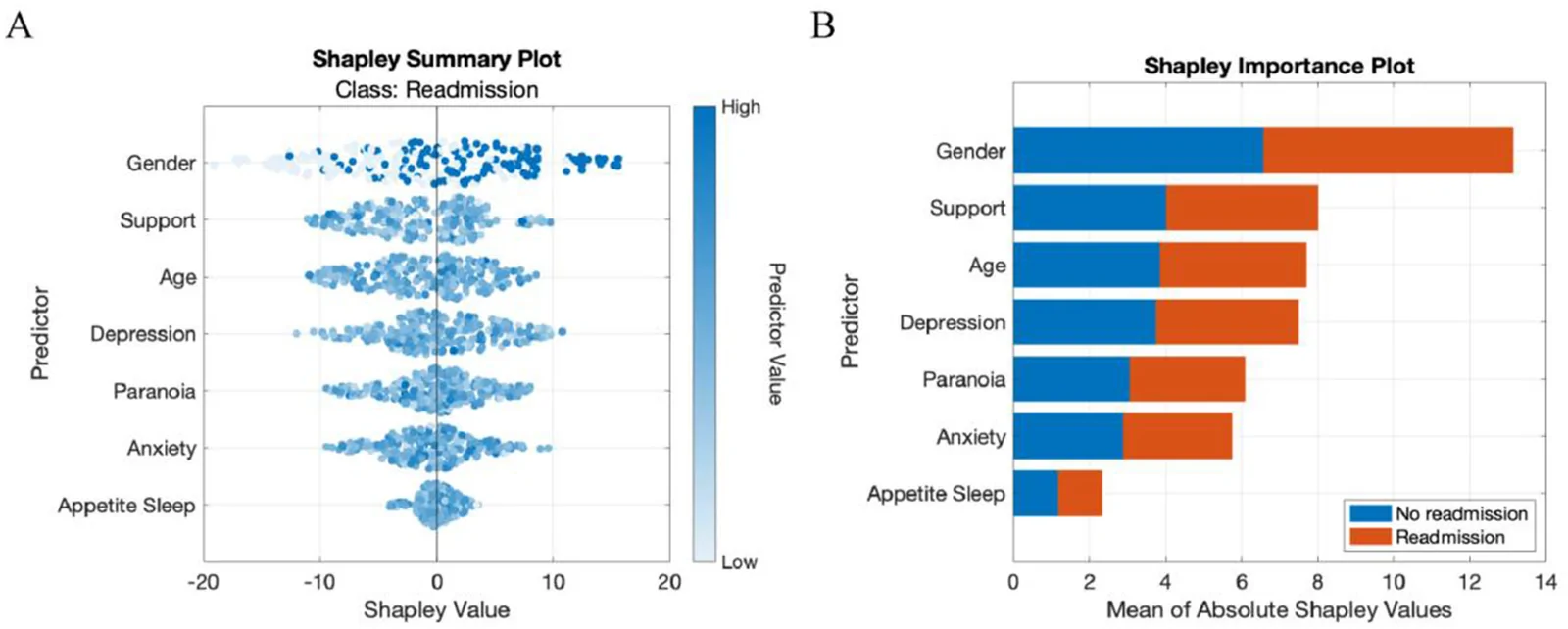
Global comparison of key indicator SHAP values. **(A)** SHAP summary plot; **(B)** SHAP feature importance plot.

**Figure 7:**
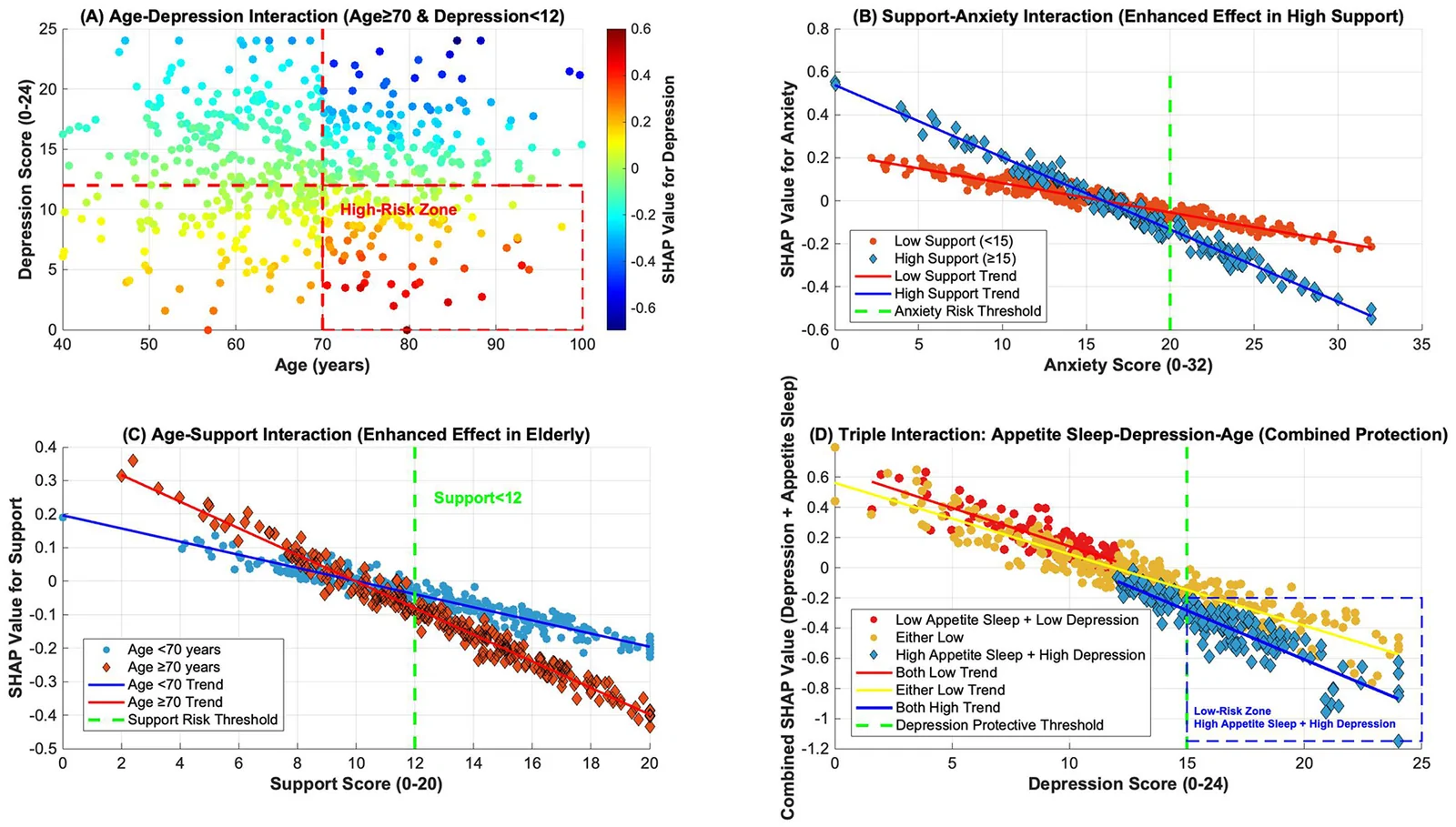
SHAP interaction analysis between key indicators. **(A)** Age-depression interaction (age 70 & depression < 12). **(B)** Support-anxiety interaction (enhanced effect in high support). **(C)** Age-support interaction (enhanced effect in elderly). **(D)** Triple interaction: appetite sleep-depression-age (combined protection).

To mitigate rare event bias and ensure the robustness of effect estimation, this study employed Firth's penalized maximum likelihood logistic regression to validate the independent predictive value of all candidate variables. For the continuous variable of age, the measurement unit was specified to facilitate clinical interpretation. The analysis confirmed that among PROM-related variables, improvements in all domain scores exhibited significant protective effects. Specifically, each one-year increase in age significantly elevated the readmission risk by 6.086-fold (OR = 6.086, 95% CI: 3.032–12.219); the readmission risk for female patients was approximately 5.892 times that of males (OR = 5.892, 95% CI: 1.829–18.976). In contrast, improvements in psychosocial domains, such as reduced depressive symptoms (OR = 0.245, 95% CI: 0.141–0.428) and decreased anxiety levels (OR = 0.338, 95% CI: 0.224–0.508), demonstrated significant protective effects independent of traditional factors (Table 6).

**Table 6:** Multivariate logistic regression analysis of patient readmission.

Variables	β	SE	Wald	*P*	OR	OR 95% CI
Gender (female vs. male)	1.774	0.596	8.857	0.003	5.892	1.829~18.976
Age (per 1-year increase)	1.806	0.354	26.031	< 0.001	6.086	3.037~12.194
Reduced depressive symptoms	−1.406	0.285	24.334	< 0.001	0.245	0.141~0.428
Reduced anxiety	−1.085	0.210	26.694	< 0.001	0.338	0.224~0.508
Enhanced social support	−0.908	0.196	21.465	< 0.001	0.403	0.274~0.592
Reduced suspiciousness	−0.599	0.137	19.114	< 0.001	0.549	0.419~0.720
Improved appetite sleep	−0.172	0.080	4.622	0.032	0.842	0.720~0.985

To eliminate response bias from reverse-worded items, all negatively worded items were standardized by directional adjustment (adjusted score = 4—raw score). Higher scores on all PROM continuous variables represent better health status in that domain, and OR values less than 1 indicate protective factors. This model included 7 primary predictors, with 141 readmission events in the training set, corresponding to approximately 20.1 events per variable. Given the sparsity of the data, Firth's penalized maximum likelihood estimation was used for robust estimation, and all effect sizes are penalized adjusted results.

### 3.7 Clinical decision support system

We developed an intuitive visualization system enabling real-time readmission risk assessment (Figure 8). Users input feature values into designated fields, and the system automatically generates risk stratification with interpretable outputs.

**Figure 8:**
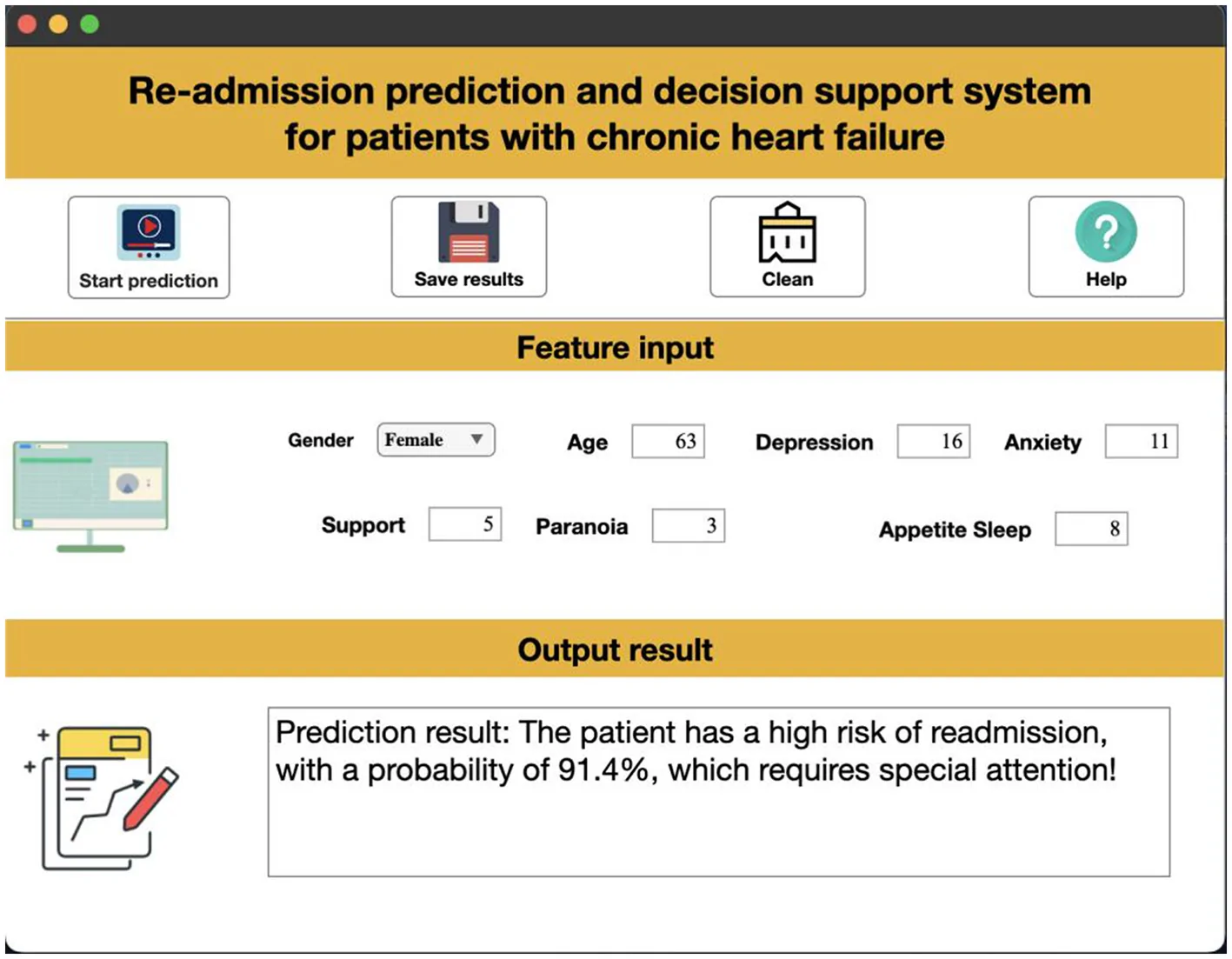
Clinical decision support system demonstration interface.

## 4 Discussion

This study innovatively integrates an AutoML framework with PROM to develop a predictive system for readmission risk in chronic heart failure (CHF) patients. Our findings reveal that psychosocial factors (depression, anxiety, social support, etc.) synergize with demographic variables to impact patient prognosis, offering a multidimensional assessment perspective for heart failure management. Model validation demonstrates the AutoML algorithm's superior predictive efficacy, while decision curve analysis showed that the model yielded net benefit across a wide range of clinical risk thresholds, but external validation is needed to confirm the clinical generalizability of this finding. The system enables dynamic risk stratification for high-risk patients through real-time outputs, aligning with growing global emphasis on patient-reported experience metrics. Notably, WHO now prioritizes health-related quality of life (HRQoL) as a core cardiovascular outcome indicator (20, 21). Our study systematically quantifies the dose-effect relationship between HRQoL improvement and reduced readmission risk, suggesting depression alleviation and enhanced social support as potential targets for reducing healthcare burdens. Clinically, our AutoML framework introduces three innovations: (1) Accessibility: Transcends laboratory-dependent models by converting PROMs into digital biomarkers deployable in community settings; (2) Actionability: Integrated CDSS prioritizes high-risk patients for targeted interventions; (3) Utility: Net benefit over traditional models across clinically relevant thresholds. Unlike static nomograms, our system continuously learns from new cases via federated learning, adapting to CHF's pathological heterogeneity.

Female sex and advanced age emerged as traditional biomedical risk factors (OR = 7.248 and 6.086, respectively), consistent with established mechanisms of physiological decline and hormonal regulation (2, 22). More significantly, PROM-derived psychological risk factors—depression, anxiety, and suspiciousness—exceeded certain clinical biomarkers in predictive strength, potentially implicating sympathetic overactivation in cardiac deterioration (23). The prominence of suspiciousness as a non-traditional risk factor contrasts with Lee et al.'s personality disorder studies (24), possibly reflecting cultural divergence: in Asian contexts, health-related hypervigilance may prompt timely care-seeking through family involvement, yet paradoxically exacerbate iatrogenic anxiety when communication falters (25). Protective factors included enhanced social support and improved appetite-sleep composite scores, with enhanced social support associated with an approximately 60% reduction in readmission risk (OR = 0.403, 95% CI: 0.274–0.592). Furthermore, the improved appetite sleep metric (OR = 0.842) underscores circadian and nutritional impacts on cardiac metabolism, supporting nocturnal oximetry-guided dietary interventions (26, 27).

The SHAP analysis revealed a multidimensional hierarchy of psychosocial predictors contributing to readmission risk. In descending order of feature importance, the key psychosocial factors identified were: depressive mood (ranked first), anxiety (ranked second), social support (ranked third), suspiciousness (ranked fourth), and appetite-sleep status (ranked fifth). Among these, SHAP dependence plots demonstrated that lower depressive symptom scores, higher social support levels, and favorable appetite-sleep scores were consistently associated with protective effects against readmission. Notably, multivariate logistic regression confirmed that reduced anxiety levels conferred a significant independent protective effect (OR = 0.338, 95% CI: 0.224–0.508), underscoring the importance of anxiety assessment beyond depression screening. The interaction analysis further revealed synergistic protective patterns: patients with high social support combined with low anxiety, and those with favorable appetite-sleep combined with low depression, exhibited disproportionately lower predicted readmission probabilities, suggesting that these psychosocial factors do not operate in isolation but rather exert combined effects that may inform combined intervention strategies. Our study extends the SHAP-based factor analysis through systematic evaluation of interaction mechanisms, revealing an anomalous risk surge in the dual-high-risk profile (age ≥70 years and reduced depressive symptomatology score < 12, indicating persistent severe depressive symptoms)—a phenomenon termed the geriatric depression pseudonormalization trap. Paradoxically low depression scores in this cohort often mask impaired self-assessment capacity stemming from cognitive decline, leading to delayed healthcare-seeking due to emotional suppression and culminating in a silent high-risk cohort. This discovery challenges unidimensional depression score interpretations and necessitates age-stratified clinical standards, advocating for cognitive correction modules within the Geriatric Depression Scale (GDS). Social support demonstrated multi-tiered protective effects: across the cohort, high support levels enhanced anxiety mitigation by buffering environmental stressors and reducing sympathetic tone, while elderly subgroups exhibited amplified protection attributable to sociocultural specificities of East Asian intergenerational family structures. Most significantly, body-mind HRQoL synergism emerged wherein the seemingly paradoxical coexistence of robust appetite-sleep metrics and elevated depression scores revealed dual-pathway autonomic regulation. This mechanism enables sufficient physiological reserves to convert depression-associated emotional hyperactivation into enhanced treatment adherence, ultimately reducing readmission risk in this subpopulation through neurobiological-psychological interplay.

The multidimensional psychosocial risk profile identified in this study aligns with and extends prior work on psychosocial predictors of heart failure readmission. Consistent with previous reports, depressive symptoms emerged as the most important psychosocial predictor of readmission risk, corroborating the well-established association between depression and adverse heart failure outcomes (28, 29). Similarly, the protective role of social support identified in our SHAP analysis resonates with longitudinal evidence demonstrating that higher perceived social support is associated with reduced hospitalization rates in heart failure populations (30). However, our study extends these findings in several important respects. First, beyond the traditionally studied factors of depression and social support, our SHAP analysis identified anxiety and suspiciousness as significant additional predictors, with anxiety ranked second in importance only to depressive mood. This finding suggests that the emotional burden in heart failure is not confined to depressive symptoms alone but encompasses a broader anxiety-depression spectrum that warrants comprehensive assessment. Second, the appetite-sleep dimension—a somatic manifestation of psychosocial distress—was identified as an important protective factor through SHAP interaction analysis, a dimension that has been largely underexplored in previous readmission prediction models. The simultaneous evaluation of 12 psychosocial subdimensions within a unified AutoML framework represents a methodological advance over prior studies that typically examined these factors in isolation, thereby enabling the identification of synergistic effects and relative importance rankings that single-domain analyses cannot capture.

The multidimensional psychosocial risk stratification framework developed in this study carries several practical implications for clinical heart failure management. First, the identification of specific psychosocial risk profiles—including the independent protective effect of reduced anxiety (OR=0.338) and the synergistic interactions between social support and depressive symptoms—enables clinicians to move beyond generic risk categorization toward personalized intervention planning. For instance, elderly female patients with concurrently elevated depressive and anxiety scores represent a high-risk subgroup that may benefit from integrated psychological support and closer post-discharge surveillance. Second, the SHAP-based interpretability framework provides individualized risk explanations that can facilitate shared decision-making at the point of care. The MATLAB-based visualized decision-making tool developed in this study translates complex machine learning outputs into clinically interpretable risk estimates, potentially lowering the barrier to adoption in routine clinical workflows. Third, the CHF-PROM-based assessment approach is relatively low-cost and non-invasive, requiring only a validated questionnaire rather than specialized laboratory testing, which supports its feasibility for widespread implementation in resource-limited settings. The integration of this psychosocial screening tool with existing disease management programs could enable a more proactive, biopsychosocially-informed care model that addresses not only hemodynamic and pharmacological management but also the psychological and social determinants of readmission risk.

## 5 Limitations and future directions

This study has several methodological limitations that should be carefully considered when interpreting the findings. First, despite rigorous cultural adaptation of the assessment instruments, 37% of patients exhibited socially desirable response bias, particularly when revising answers to sensitive items such as those concerning depressive symptoms. This pattern of responding may compromise the accuracy of self-reported psychosocial data and introduces potential measurement bias. Second, the sample was drawn from a single-center retrospective dataset, and a 23.7% loss-to-follow-up rate was observed among NYHA class IV patients, largely attributable to physical or cognitive impairments that precluded their participation. This attrition raises concerns about survivor bias and limits the representativeness of the most severely ill heart failure population. Third, the current dataset lacks biological granularity, notably the absence of pharmacogenomic profiles (e.g., CYP2D6 phenotypes) and gut microbiome data, which constrains the depth of biopsychosocial pathway exploration possible within this framework.

To address these limitations, several research directions are planned. Regarding the issue of socially desirable responding, future iterations of the framework may integrate voice-affective analysis utilizing a CNN-LSTM architecture to capture micro-emotional fluctuations that are less susceptible to conscious control, alongside wearable-derived heart rate variability data to establish autonomic-emotional cross-mapping. To improve sample representativity and mitigate survivor bias, solutions currently under development include ICU-compatible dynamic patient-reported outcome measures incorporating eye-tracking selection mechanisms for patients with severe physical limitations, caregiver proxy assessment modules equipped with reliability scoring algorithms, and transitional care monitoring systems designed to track post-discharge vital sign fluctuations during the critical early discharge period. To enhance biological granularity, future work will incorporate metabolomics data with the aim of constructing a “microbiome-gut-brain-heart” quadra-dimensional module capable of elucidating the complex interplay between biological and psychosocial factors. Furthermore, given the single-center nature of the current training data, a federated learning combined with transfer learning dual architecture will be explored to enable multi-institutional validation and community deployment while preserving data privacy. Finally, the temporal validity of the predictive models will be strengthened through longitudinal calibration using digital biomarkers derived from real-world data sources.

## 6 Conclusion

This system demonstrates the feasibility of integrating PROMs into an AutoML-driven predictive framework for heart failure readmission risk. The incorporation of patient-reported psychosocial features alongside clinical variables supports the multifactorial nature of readmission risk. Future work should focus on: (1) multicenter external validation to assess generalizability; (2) integration of multi-omics data (e.g., metabolomics, gut microbiome) to further explore biopsychosocial mechanisms; and (3) evaluation of framework generalizability through distributed learning architectures. As this framework continues to evolve, it may contribute to a shift from reactive symptom management toward more proactive, data-informed care strategies.

## Data Availability

The raw data supporting the conclusions of this article will be made available by the authors, without undue reservation.

## References

[1] Heidenreich PA Bozkurt B Aguilar D Allen LA Byun JJ Colvin MM . 2022 AHA/ACC/HFSA guideline for the management of heart failure: a report of the American college of cardiology/American heart association joint committee on clinical practice guidelines. Circulation. (2022) 14:e895–e1032. doi: 10.1161/CIR.0000000000001063 35363499

[2] McDonagh TA Metra M Adamo M Gardner RS Baumbach A Böhm M . 2023 Focused Update of the 2021 ESC Guidelines for the diagnosis and treatment of acute and chronic heart failure. Eur Heart J. (2023) 44:3627–39. doi: 10.15829/1560-4071-2023-516838410903

[3] Szlagor M Dybiec J Młynarska E Rysz J Franczyk B. Chronic kidney disease as a comorbidity in heart failure. Int J Mol Sci. (2023) 24:2988. doi: 10.3390/ijms24032988 36769308 PMC9918100

[4] Bekelman DB Feser W Morgan B Welsh CH Parsons EC Paden G . Nurse and social worker palliative telecare team and quality of life in patients with COPD, heart failure, or interstitial lung disease: the ADAPT randomized clinical Trial. JAMA. (2024) 331:212–23. doi: 10.1001/jama.2023.24035 38227034 PMC10792473

[5] Molloy C Long L Mordi IR Bridges C Sagar VA Davies EJ . Exercise-based cardiac rehabilitation for adults with heart failure. Cochrane Database Syst Rev. (2024) 3:CD003331. doi: 10.1002/14651858.CD003331.pub6 38451843 PMC10919451

[6] Hai AA Weiner MG Livshits A Brown JR Paranjape A Hwang W . Domain generalization for enhanced predictions of hospital readmission on unseen domains among patients with diabetes. Artif Intell Med. (2024) 158:103010. doi: 10.1016/j.artmed.2024.103010 39556977 PMC11602339

[7] Brown JR Ricket IM Reeves RM Shah RU Goodrich CA Gobbel G . Information extraction from electronic health records to predict readmission following acute myocardial infarction: does natural language processing using clinical notes improve prediction of readmission? J Am Heart Assoc. (2022) 11:e024198. doi: 10.1161/JAHA.121.024198 35322668 PMC9075435

[8] Verma VK Lin WY. Machine Learning-based 30-day hospital readmission predictions for COPD patients using physical activity data of daily living with accelerometer-based device. Biosensors. (2022) 12:605. doi: 10.3390/bios12080605 36005000 PMC9406028

[9] Matheny ME Ricket I Goodrich CA Shah RU Stabler ME Perkins AM . Development of electronic health record-based prediction models for 30-day readmission risk among patients hospitalized for acute myocardial infarction. JAMA Netw Open. (2021) 4:e2035782. doi: 10.1001/jamanetworkopen.2020.35782 33512518 PMC7846941

[10] Petty MG Wu T Andrei AC Baldridge A Warzecha A Kao A . Baseline quality-of-life of caregivers of patients with heart failure prior to advanced therapies: findings from the sustaining quality of life of the aged: transplant or mechanical support (SUSTAIN-IT) study. J Card Fail. (2022) 28:1137–48. doi: 10.1016/j.cardfail.2022.03.358 35470057 PMC10010287

[11] Lawson CA Benson L Squire I Zaccardi F Ali M Hand S . Changing health related quality of life and outcomes in heart failure by age, sex and subtype. EClinicalMedicine. (2023) 64:102217. doi: 10.1016/j.eclinm.2023.102217 37745020 PMC10514432

[12] Johansson I Joseph P Balasubramanian K McMurray JJV Lund LH Ezekowitz JA . Health-Related quality of life and mortality in heart failure: the global congestive heart failure study of 23 000 patients from 40 countries. Circulation. (2021) 143:2129–42. doi: 10.1161/CIRCULATIONAHA.120.050850 33906372

[13] Francis J Prothasis S George J Stoica S. Patient-reported outcome measures in congenital heart surgery: a systematic review. Cardiol Young. (2023) 3:337–41. doi: 10.1017/S1047951123000057 36650739

[14] Tian J Xue J Hu X Han Q Zhang Y. CHF-PROM: validation of a patient-reported outcome measure for patients with chronic heart failure. Health Qual Life Outcomes. (2018) 1:51. doi: 10.1186/s12955-018-0874-2 29554963 PMC5859646

[15] Deger SU. A study on heart data analysis and prediction using advanced machine learning methods. Comput Biol Med. (2025) 192:110308. doi: 10.1016/j.compbiomed.2025.110308 40359679

[16] Wang X Mu X Li X Yang C Cai Y Liu C . Construction of a deep learning model and identification of the pivotal characteristics of FGF7- and MGST1- positive fibroblasts in heart failure post-myocardial infarction. Int J Biol Macromol. (2025) 310:143171. doi: 10.1016/j.ijbiomac.2025.143171 40258553

[17] Chalati MD Shirvankar C Gore G Sharma A Abbasgholizadeh-Rahimi S. Are Machine Learning methods effective in detecting undiagnosed atrial fibrillation in primary care settings using electronic health records? A systematic review. PLOS Digit Health. (2025) 4:e0001009. doi: 10.1371/journal.pdig.0001009 41086202 PMC12520348

[18] Khalsa SSS Hollon TC Adapa A Urias E Srinivasan S Jairath N . Automated histologic diagnosis of CNS tumors with machine learning. CNS Oncol. (2020) 9:CNS56. doi: 10.2217/cns-2020-0003 32602745 PMC7341168

[19] Bouaouda. A, Hashim. F.A, Sayouti. Y, et al. Pied kingfisher optimizer: a new bio-inspired algorithm for solving numerical optimization and industrial engineering problems. Neural Computing Applications. (2024) 3:15455–13. doi: 10.1007/s00521-024-09879-5

[20] Han Q Ren J Tian J Yang H Zhang Q Wang R . A nomogram based on a patient-reported outcomes measure: predicting the risk of readmission for patients with chronic heart failure. Health Qual Life Outcomes. (2020) 1:290. doi: 10.1186/s12955-020-01534-6 32854729 PMC7450976

[21] Frampton JE. Empagliflozin: A review in symptomatic chronic heart failure. Drugs. (2022) 8:1591–602. doi: 10.1007/s40265-022-01778-0 36374374

[22] Di Palo KE Barone NJ. Hypertension and heart failure: prevention, targets, and treatment. Cardiol Clin. (2022) 4:237–44. doi: 10.1016/j.ccl.2021.12.011 35465897

[23] Cui X Zhou X Ma LL Sun TW Bishop L Gardiner FW . A nurse-led structured education program improves self-management skills and reduces hospital readmissions in patients with chronic heart failure: a randomized and controlled trial in China. Rural Remote Health. (2019) 1:5270. doi: 10.22605/RRH5270 31113205

[24] Lee HB Bienvenu OJ Cho SJ Ramsey CM Bandeen-Roche K Eaton WW . Personality disorders and traits as predictors of incident cardiovascular disease: findings from the 23-year follow-up of the Baltimore ECA study. Psychosomatics. (2010) 5:289–96. doi: 10.1016/S0033-3182(10)70699-X 20587756 PMC4086910

[25] Aronow WS Shamliyan TA. Comparative effectiveness of disease management with information communication technology for preventing hospitalization and readmission in adults with chronic congestive heart failure. J Am Med Dir Assoc. (2018) 1:472–9. doi: 10.1016/j.jamda.2018.03.012 29730178

[26] Hale TM Jethwani K Kandola MS Saldana F Kvedar JC. A Remote Medication Monitoring System for Chronic Heart Failure Patients to Reduce Readmissions: a Two-Arm Randomized Pilot Study. J Med Internet Res. (2016) 1:e91. doi: 10.2196/jmir.5256 27154462 PMC4890732

[27] Yamamoto S Okamura M Akashi YJ Tanaka S Shimizu M Tsuchikawa Y . Impact of Long-term exercise-based cardiac rehabilitation in patients with chronic heart failure-a systematic review and meta-analysis. Circ J. (2024) 8:1360–71. doi: 10.1253/circj.CJ-23-0820 38220206

[28] Zhao H Jiang J Zhong G Peng Y Wen Y Liang Y . Prognostic value of depressive symptoms for cardiovascular events in female patients with heart failure with reduced ejection fraction and heart failure with preserved ejection fraction. J Am Heart Assoc. (2024) 13:e032961. doi: 10.1161/JAHA.123.032961 38686893 PMC11179909

[29] Friedrich ME Perera G Leutgeb L Haardt D Frey R Stewart R . Predictors of hospital readmission for patients diagnosed with delirium: an electronic health record data analysis. Acta Psychiatr Scand. (2023) 147:506–15. doi: 10.1111/acps.13523 36441117 PMC10463092

[30] Sterling MR Ringel JB Pinheiro LC Safford MM Levitan EB Phillips E . Social determinants of health and 30-day readmissions among adults hospitalized for heart failure in the REGARDS study. Circ Heart Fail. (2022) 15:e008409. doi: 10.1161/CIRCHEARTFAILURE.121.008409 34865525 PMC8849604

