# Supplementary material for: Construction of a readmission prediction model and development of a decision support system for chronic heart failure patients based on patient-reported outcome measures
Source: Front Med (Lausanne). 2026 Sep 23;13:1748709. doi: 10.3389/fmed.2026.1748709 (PMC13643751; doi:10.3389/fmed.2026.1748709)
Supplement: Supplementary file 1 [file Table_1.docx]

**Algorithm: Improved Pied Kingfisher Optimizer (IPKO)**

function [best_sol, best_fit] = IPKO(pop_size, max_iter, dim, lb, ub)

% Algorithm: Improved Pied Kingfisher Optimizer (IPKO)

% Inputs: pop_size - population size

% max_iter - maximum iterations

% dim - problem dimension

% lb, ub - lower/upper bounds (vectors)

% Outputs: best_sol - best solution found

% best_fit - corresponding fitness value

% ---------- 1. Sinusoidal chaotic initialization ----------

pos = zeros(pop_size, dim);

for i = 1:pop_size

for j = 1:dim

chaotic_val = sin(pi * rand()); % sinusoidal mapping

pos(i,j) = lb(j) + chaotic_val * (ub(j) - lb(j));

end

end

fit = zeros(pop_size, 1);

for i = 1:pop_size

fit(i) = objective_function(pos(i,:));

end

[best_fit, idx] = min(fit);

best_sol = pos(idx,:);

best_fit_old = best_fit;

% ---------- 2. Main optimization loop ----------

for t = 1:max_iter

a = 2 * (1 - t/max_iter)^(1/3); % nonlinear convergence factor

for i = 1:pop_size

if rand() < 0.5

% Hovering search (standard PKO) + dynamic Gaussian walk

gaussian_step = a * (1 - t/max_iter) * randn(1, dim);

new_pos = standard_PKO_hovering(pos(i,:), best_sol, pos, gaussian_step);

else

% Diving prey-catching (standard PKO) with Levy flight

new_pos = standard_PKO_diving(pos(i,:), best_sol);

end

% Boundary reflection

new_pos = min(max(new_pos, lb), ub);

% Greedy selection

new_fit = objective_function(new_pos);

if new_fit < fit(i)

pos(i,:) = new_pos;

fit(i) = new_fit;

if new_fit < best_fit

best_fit = new_fit;

best_sol = new_pos;

end

end

end

% ---------- 3. Stagnation handling: chaotic reset ----------

if best_fit >= best_fit_old % no improvement in this iteration

[~, sort_idx] = sort(fit);

worst_idx = sort_idx(ceil(0.8*pop_size):end);

for w = worst_idx'

for j = 1:dim

chaotic_val = sin(pi * rand());

pos(w,j) = lb(j) + chaotic_val * (ub(j) - lb(j));

end

fit(w) = objective_function(pos(w,:));

end

end

best_fit_old = best_fit;

end

end
